# Cancer Stem Cells as a Potential Target to Overcome Multidrug Resistance

**DOI:** 10.3389/fonc.2020.00764

**Published:** 2020-06-02

**Authors:** Yena Cho, Yong Kee Kim

**Affiliations:** Research Institute of Pharmaceutical Sciences, College of Pharmacy, Sookmyung Women's University, Seoul, South Korea

**Keywords:** multidrug resistance, ABC transporters, P-glycoprotein, cancer stem cells, epigenetics

## Abstract

Multidrug resistance (MDR), which is a significant impediment to the success of cancer chemotherapy, is attributable to various defensive mechanisms in cancer. Initially, overexpression of ATP-binding cassette (ABC) transporters such as P-glycoprotein (P-gp) was considered the most important mechanism for drug resistance; hence, many investigators for a long time focused on the development of specific ABC transporter inhibitors. However, to date their efforts have failed to develop a clinically applicable drug, leaving only a number of problems. The concept of cancer stem cells (CSCs) has provided new directions for both cancer and MDR research. MDR is known to be one of the most important features of CSCs and thus plays a crucial role in cancer recurrence and exacerbation. Therefore, in recent years, research targeting CSCs has been increasing rapidly in search of an effective cancer treatment. Here, we review the drugs that have been studied and developed to overcome MDR and CSCs, and discuss the limitations and future perspectives.

## Introduction

Chemotherapy is one of the most effective treatments for cancer; however, its success has been challenged by the acquisition of multidrug resistance (MDR) (1). MDR is caused by sustained (dose-dependent or time-dependent) administration of chemotherapeutic drugs, resulting in cross-resistance to a broad spectrum of structurally- and mechanically-distinct chemotherapeutic drugs (2). There are several mechanisms underlying MDR (3, 4): (i) increased pumping out of the drug through efflux pumps such as P-glycoprotein (P-gp) encoded by *ABCB1* (5); (ii) decreased uptake of the drug through transporters; (iii) activation of drug-metabolizing enzymes such as cytochrome P450 and glutathione S-transferase; (iv) activation of DNA repair systems; (v) evasion of apoptosis. The first three of these processes are conducive to the development of resistance by preventing the drug from reaching an effective concentration, while the remaining two mechanisms achieve resistance by detoxifying the action of the drug. Based on the mechanisms described, we have been trying to establish strategies for overcoming MDR. Before discussing them further, we need to understand the existence of cancer stem cells (CSCs) and their roles in cancer biology. CSCs, also known as tumor-initiating cells (TICs), are a small population of cancer cells that have the ability to self-renew and differentiate similar to normal stem cells (NSCs). However, as CSCs are tumorigenic, they can contribute to the aggravation and recurrence of cancer (6). According to a CSC model that explains the relationship between CSC and MDR, increased expression of ATP-binding cassette (ABC) transporters and other genes contributes to the intrinsic resistance of CSCs to chemotherapy (7, 8). CSCs have relatively slow cell-cycle kinetics and are therefore targeted less by chemotherapeutic drugs compared to rapidly dividing cells (9). In addition to the well-known ABC transporters (2, 10), many other drug resistance mechanisms of CSCs have been identified, for example, aldehyde dehydrogenases (ALDHs) (11), epithelial-mesenchymal transition (EMT) (12), epigenetic modifications (13, 14), factors affecting tumor microenvironment, such as hypoxia (15), and signaling pathways (16–18). In this review, we provide a brief outline of MDR mechanisms and focus on the investigated drugs.

## Current Strategies to Overcome MDR

### Targeting ABC Transporters

ABC transporters including *ABCB1, ABCC1*, and *ABCG2* are expressed in cancer stem/progenitor cells. These transporters have broad drug specificity and pump out a wide range of structurally- and mechanically-unrelated compounds, thereby lowering the intracellular accumulation of these compounds and consequently diminishing their biological efficacies (19). Several chemotherapeutic agents in clinical use are susceptible to ABC transporter-mediated efflux, such as microtubule-targeting taxanes (e.g., docetaxel and paclitaxel) and vinca alkaloids (vinblastine and vincristine), DNA-damaging anthracyclines (daunorubicin and doxorubicin), topoisomerase inhibitors (etoposide and topotecan), and tyrosine kinase inhibitors (dasatinib and gefitinib) (20). Therefore, developing strategies to target ABC transporters is an important area of cancer research, and many studies have been conducted accordingly (21). There are three approaches: (i) regulating the function of ABC transporters using competitive or allosteric inhibitors (Table 1) as well as the antibodies that target ABC transporters, such as UIC2 and MRK16 (86); (ii) regulating gene expression of ABC transporters at the transcriptional or translational level because, as with trabectedin, it is an attractive strategy to control ABC transporters at the transcriptional level by affecting the MDR enhanceosome (87–89); (iii) using anticancer drugs that are poor substrates of P-gp, such as ixabepilone (Table 1). Until ixabepilone was launched, efforts to develop drugs targeting ABC transporters had been a driving force in the development of first-, second-, and third-generation P-gp inhibitors (90, 91). However, it has been reported that first-generation inhibitors have low potency and high toxicity, and second-generation inhibitors have frequent drug-drug interactions (92). With the third-generation inhibitors, there have been many improvements with regard to the drawbacks of the previous generations, but clinical trial data are still insufficient. In effect, most clinical trials have been discontinued. Because NSCs, including hematopoietic stem cells, unrestricted somatic stem cells, and mesenchymal stem cells, also express ABC transporters to protect themselves from cytotoxic agents (93), inhibiting ABC transporters may cause serious side effects such as hematopoietic disorders due to bone marrow dysfunction. Therefore, the emergence of ixabepilone was inevitable and has been well-received. Like taxanes, ixabepilone leads to G_2_/M phase arrest by stabilizing microtubules and promoting tubulin polymerization. However, ixabepilone has a very important feature (not found in taxanes) effective against cancer cells that acquire MDR following repeated chemotherapy, as this drug is not pumped out through P-gp. Now, developing drugs that are not substrates of P-gp has become a trend for overcoming MDR cancer. In light of ixabepilone, chemical modifications of paclitaxel and vinblastine have also been attempted in succession, producing cabazitaxel and ortataxel, and vinflunine, respectively (Table 1). After these modifications, increased cytotoxic effects were observed in P-gp-overexpressing cell lines (43).

**Table 1:** Drugs that reverse chemoresistance via various mechanisms.

**Drugs**	**MOA**	**Clinical trial**	**References**
**ABC transporter inhibitors**			
Cyclosporine	1st generation ABCB1 competitive inhibitors	–	(22)
Nicardipine			(23)
Quinine			(24)
Tamoxifen			(25)
Verapamil			(26)
Cinchonine	2nd generation ABCB1 competitive inhibitors	–	(27)
Dexverapamil			(28)
Toremifene			(29)
Valspodar (PSC-833)			(30)
Biricodar (VX-710)	2nd generation ABCB1 and ABCC1 competitive inhibitors	–	(31)
Dofequidar (MS-209)			(32)
Laniquidar (R101933)	3rd generation ABCB1 competitive inhibitors	–	(33)
Lonafarnib (SCH66336)			(34)
Zosuquidar (LY335979)			(35)
Elacridar (GF120918)	3rd generation ABCB1 and ABCG2 competitive inhibitors	–	(36)
Tariquidar (XR9576)			(37)
Fumitremorgin C	ABCG2 competitive inhibitor	–	(38)
Flupentixol	ABCB1 allosteric modulators	–	(39)
**Non-substrates of P-gp**			
Ixabepilone	Microtubule inhibitor	FDA approval	(40)
Cabazitaxel			(41)
Ortataxel		Phase 2	(42)
Vinflunine		Phase 3	(43)
**ALDH1 inhibitors**			
Disulfiram	Copper-dependent proteasome inhibitor	Phase 2	(44, 45)
Tretinoin	–	–	(46)
**AMPK activators**			
Metformin	–	Phase 2/3	(47, 48)
Thalidezine	Autophagic cell death inducer	–	(49)
**VEGF inhibitor**			
Bevacizumab	Anti-VEGF antibody	FDA approval	(50)
**RTK inhibitor**			
Sorafenib	Multikinase inhibitor	FDA approval	(51)
**Vascular disrupting agents**			
Combretastatin A4	Tubulin-binding agents	Phase 1/2	(52)
Plocabulin			(53)
**BET inhibitors**			
TEN-010 (JQ2)	–	Phase 1	(54)
CC-90010	BRD2 inhibitor	Phase 1	(54)
ABBV-744	BRD4 inhibitors	Phase 1	(55)
CPI-0610			(56)
I-BET151	BRD2 and BRD4 inhibitor	Phase 1	(57)
OTX015	BRD2, BRD3, and BRD4 inhibitor	Phase 1	(58, 59)
ABBV-075	BRD2, BRD3, BRD4, and BRDT inhibitors	Phase 1	(60)
FT-1101			(54)
I-BET762		Phase 1/2	(61)
**HDAC inhibitors**			
Vorinostat	Pan-HDAC inhibitors	FDA approval	(62, 63)
Panobinostat			(64)
**KDM1 inhibitors**			
Tranylcypromine	Non-selective irreversible inhibitor	Phase 1/2	(65)
GSK-2879552	Selective irreversible inhibitors	Phase 1	(66)
IMG-7289 (Bomedemstat)		Phase 1/2	(67)
INCB-059872			(68)
ORY-1001 (Iadademstat)			(69)
CC-90011	Selective reversible inhibitors	Phase 1	(70)
SP−2577 (Seclidemstat)		Phase 1/2	(71)
**Notch signaling inhibitors**			
DAPT	γ-secretase inhibitors	–	(72, 73)
RO4929097		Phase 2	(74)
**Hh signaling inhibitors**			
Cyclopamine	Smo inhibitors	–	(75, 76)
Sonidegib		FDA approval	(77)
Vismodegib			(77)
**Wnt/β-catenin signaling inhibitors**			
Ipafricept	FZD8-Fc	Phase 1	(78)
Rosmantuzumab	Anti-RSPO antibody		(79)
Vantictumab	Anti-FZD1, 2, 5, 7, and 8 antibody		(79, 80)
Foxy-5	Wnt5a-mimicking peptide	Phase 2	(81)
ETC-159	Porcupine inhibitors	Phase 1	(82)
LGK974		Phase 2	(83)
CWP232291	β-catenin inhibitors	Phase 1	(84)
PRI-724			(85)

### Targeting Aldehyde Dehydrogenases

ALDH plays an important role in the differentiation of stem cells by converting retinol into retinoic acid, as well as detoxifying the cells by converting aldehyde into carboxylic acid, and thus ALDH is considered a biomarker for stem cells (11, 94). Several studies have shown that ALDH-1 is correlated with CSCs. Gefitinib, an EGFR inhibitor, is used for breast-, lung-, and other cancers. However, it has been confirmed that ALDH1A1-positive CSCs are more resistant to gefitinib than ALDH1A1-negative CSCs (95). ALDH-1 expression is mediated by high expression of Snail, which regulates metastasis as a transcription factor and subsequently causes CSCs to develop resistance to chemotherapy (96). To solve this problem, ALDH inhibitors, such as diethylaminobenzaldehyde, disulfiram, and tretinoin, have been proposed (Table 1). Bromodomain and extra-terminal (BET) inhibitors such as JQ1 have also been proposed due to their ability to suppress ALDH1A1 expression (97).

### Targeting Epithelial-Mesenchymal Transition

EMT is a process by which epithelial cells become mesenchymal stem cells. In this process, epithelial cells lose cell polarity and cell-cell adhesion function but gain migratory and invasive functions (98, 99). In other words, EMT causes cancer cells to exhibit stem-like features such as tumorigenicity. EMT also promotes metastasis that occurs mainly due to reduced expression of E-cadherin, which itself is directly repressed by Snail (100). Because EMT plays a crucial role in chemoresistance of CSCs, strategies to inhibit this process can be effective. Some studies have shown that AMP-activated kinase (AMPK) induces apoptosis of cancer cells, inhibits TGF-β-induced EMT and, consequently, can reverse drug resistance (101). AMPK activators that have been proposed include metformin and thalidezine (Table 1). Although the data are still insufficient, these activators seem to have enough potential to overcome chemoresistance. It has also been proposed that histone deacetylase (HDAC) inhibitors suppress EMT and attenuate chemoresistance (102).

### Targeting Epigenetic Modifications

Histone acetylation, one of the most common post-translational modifications, is closely associated with CSC chemoresistance. This process, in which lysine residues are acetylated, is tightly regulated by histone acetyltransferases (HATs) and HDACs. Bromodomains (BRD) of the BET family proteins read acetyl-lysine (Kac) residues on histones and regulate gene expression. BET family proteins recruit the positive transcriptional elongation factor (P-TEFb), resulting in a transcriptional cascade of oncogenes (54). Thus, inhibition of BET binding to acetylated histones suppresses cell proliferation and induces apoptosis. JQ1 is a potent, highly specific, and Kac competitive inhibitor for BET family proteins (97). Because JQ1 has a short half-life, its derivatives and structurally similar forms are undergoing clinical trials (Table 1). On the other hand, HDACs, which are epigenetic erasers, induce stem-like features by promoting EMT. HDACs also affect hypoxia-inducible factors (HIFs) and NF-κB related to apoptosis (103, 104), which are components of the tumor microenvironment. HDAC inhibitors, including vorinostat (SAHA) and panobinostat, suppress EGFR expression and reverse EMT (Table 1). In addition, these drugs have been successfully used in combination with BET inhibitors. Such epigenetic combination therapy improves clinical efficacy by reducing *Myc* expression. Lysine-specific demethylase 1 (KDM1, also known as LSD1) modulates histone methylation, and demethylation of H3K9me2, H3K4me3, and H3K36me3 contributes to KDM1-mediated chemoresistance (105). Inhibition of KDM1 activity was initially observed in monoamine oxidase (MAO) inhibitors such as tranylcypromine, which was accompanied by suppression of the stem cell properties of CSCs *in vivo* (106). Based on their structure, several selective KDM1 inhibitors have been developed (Table 1). In addition, the activity of KDM1 can be regulated by HDAC inhibitors, as crosstalk exists between KDM1 and HDAC. KDM1 and HDAC1/2 form the CoREST complex, which is associated with silencing gene expression. As a result, combination therapy with KDM1 inhibitors and HDAC inhibitors has been expected to have synergistic effects and has often been evaluated. Recently, KDM1-HDAC dual inhibitors such as corin have been reported (107). BMI1 and EZH2, which induce epigenetic silencing as polycomb group (PcG) members, have been reported to be associated with chemoresistance (108, 109). Besides, tumor suppressor genes are silenced by hypermethylation of promoter regions of DNA, and thus, chemotherapy loses its efficacy against many cancers (110).

### Targeting Microenvironment

The cellular microenvironment plays an important role in determining cellular behavior. NSCs and CSCs are generated, maintained, and regulated within this microenvironment. The tumor microenvironment (TME) creates a niche for itself that influences not only the proliferation and differentiation of CSCs but also the response to drugs (111). Although cancer-associated fibroblasts (CAFs) as well as inflammation and immune cells are components of the TME, we have to discuss the crucial role of hypoxia in the TME. Hypoxia signaling contributes to chemoresistance of CSCs by increasing the expression of ABC transporters and ALDH (8, 112, 113). In solid tumors, hypoxic regions are necessarily present and lead to angiogenesis through *HIF1A* and VEGF (114, 115). However, tumor angiogenesis is sloppy; hence, drugs do not reach effective concentrations in hypoxic cells. Thus, VEGF inhibitors such as bevacizumab and receptor tyrosine kinase (RTK) inhibitors such as sorafenib can enhance chemosensitivity. Vascular disrupting agents (VDAs), including tubulin-binding agents such as combretastatin A4 and plocabulin, also enhance chemosensitivity by increasing vessel permeability (Table 1). Of course, we should be careful not to inject these agents prior to chemotherapy because it could impair the delivery of chemotherapeutic drugs by reducing blood supply.

### Targeting Signaling Pathways

Signaling pathways control cell responses, particularly the self-renewal, differentiation, and survival of CSCs. Among them, the Notch, Hedgehog (Hh), and Wnt/β-catenin signaling pathways are responsible for drug resistance (116). Notch induces the expression of survivin, which is an anti-apoptotic gene that inhibits apoptosis. This signaling is suppressed by γ-secretase inhibitors (GSIs) such as DAPT and RO4929097, which block the second cleavage of Notch receptors and release of the Notch-IC fragment from the cell membrane (Table 1). Hh signaling is critical in embryogenesis and has been found in many cancers (75, 117). This signaling induces the activation of Smo and Gli1, which are involved in the drug resistance caused by overexpression of P-gp and BCRP (118). Thus, Smo inhibitors such as cyclopamine, vismodegib, and sonidegib can suppress chemoresistance (Table 1). Wnt/β-catenin signaling activated by Frizzleds (FZDs) is also associated with drug resistance due to overexpression of P-gp, BCRP, and MRP (119). Besides, this pathway promotes cell cycling, inhibits apoptosis, and mediates DNA repair processes. Although it has been suggested that the Wnt/β-catenin signaling pathway is undruggable, strategies to target it have been explored because of their various therapeutic potential. Indeed, there has been an effort to develop drugs that inhibit Wnt/β-catenin signaling (Table 1).

## Future Perspectives

To date, many chemotherapeutic drugs have been developed and many studies conducted to overcome MDR. The newer strategies that we have covered above have limitations, however, and so more work is needed with different approaches being explored to find effective and lasting treatment. Any strategy for overcoming MDR should not affect NCSs, but only CSCs. There are a few things to pay attention to for selective targeting of CSCs: (i) encapsulation of anticancer drugs in liposomes, micelles, and nanoparticles, i.e., nanotechnology-based drug delivery (120). The improvement of pharmacokinetic properties, tumor-specific delivery due to the enhanced permeability and retention (EPR) effect, and resistance to efflux pumps because of the size-exclusion effect are representative advantages of nanomaterials (NMs) (121–123). Thus, NMs are expected to increase the therapeutic effect and reduce unwanted side effects of anticancer drugs. Moreover, dual drug-loaded NMs can lead to better therapeutic effects (124). For example, pluronics, which are amphiphilic polymers and form nano-sized micellar structures, have been increasingly regarded as CSC modulators (125, 126). Pluronics are not just drug delivery carriers; rather, they inhibit metastasis and activate apoptosis by mediating the release of cytochrome c and apoptosis inducing factor (AIF). In addition, they can alter the microenvironment and suppress CSCs effectively. At present, only SP1049C (doxorubicin with pluronics L61 and F127 micelles) is in phase 3 trials (127), but there are reasons to be optimistic. (ii) Targeting microRNAs (miRNAs), which mediate translational repression and mRNA degradation mainly by binding to the 3′ UTR. Previous studies have shown that miRNAs regulate ABC transporters in CSCs (128, 129); for instance, miR-212, miR-328, miR-451, and so on. Some miRNAs, including miR-21 and miR-222, are oncogenic and up-regulated in cancer cells, while other miRNAs, including miR-15 and miR-181, are tumor suppressive and down-regulated in cancer cells (29, 130). (iii) Targeting MDR mRNA (131, 132), through which antisense oligonucleotides (aODNs) are used to down-regulate the expression of specific genes. Small interfering RNAs (siRNAs) also silence specific genes because they are artificial double-stranded RNA (dsRNA) molecules with functions similar to those of miRNA. If they selectively target tumorigenic genes, cancer cells will become chemosensitive. There are no clinical data yet to suggest that MDR can be completely reversed using this RNA interference (RNAi) technology, but it is likely that drugs related to RNAi will be developed in the near future. (iv) Transferring MDR genes into NSCs, especially bone marrow stem cells (133–135). Most chemotherapeutic drugs suppress the bone marrow and, as a result, blood cells such as erythrocytes, leukocytes, and thrombocytes do not function properly, and the ensuing loss of oxygen transport, immune response, and bleeding control functions causes serious adverse effects. To prevent these adverse effects, gene transfer technology has emerged, making it possible to administer high doses of chemotherapeutic drugs. In the transplantation model of CD34-positive peripheral blood stem cell (PBSC) infection with retroviral vectors containing MDR genes, long-term myeloprotection was achieved, demonstrating the safety of transplantation (136).

## Conclusion

**Figure 1:**
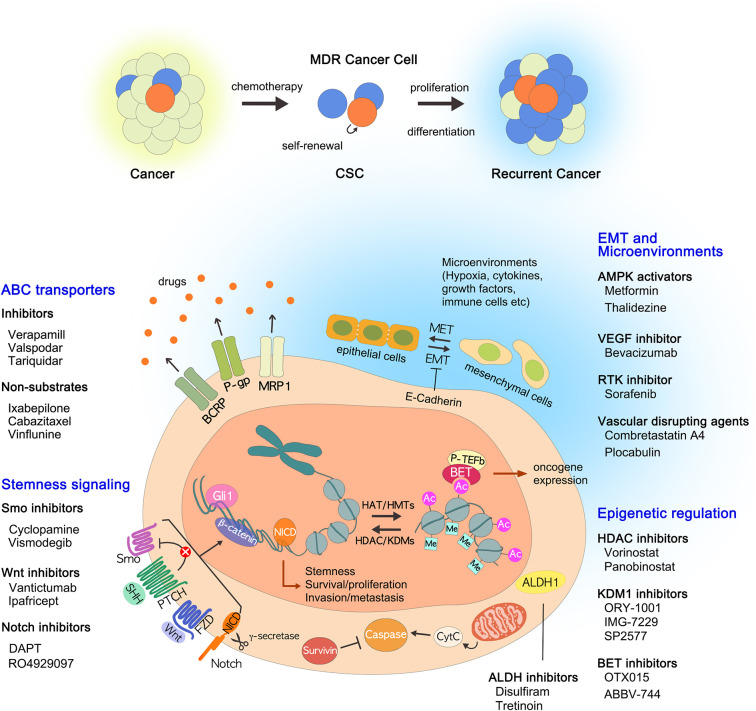
Current strategies to overcome multidrug resistance. In addition to the well-known ABC transporters, many drug resistance mechanisms of CSCs have been identified, including ALDHs, EMT, epigenetic changes, tumor microenvironment, and stemness-related signaling pathways. The drugs that inhibit each pathway are described: (i) ABC transporter inhibitors and non-substrates; (ii) ALDH inhibitors; (iii) AMPK activators; (iv) BET inhibitors, HDAC inhibitors, and KDM1 inhibitors; (v) VEGF inhibitors, RTK inhibitors, and vascular disrupting agents; (VI) Notch inhibitors, Smo inhibitors, and Wnt/β-catenin inhibitors. ABC, ATP binding cassette; ALDHs, aldehyde dehydrogenases; AMPK, AMP-activated kinase; BET, bromodomain and extra-terminal; CSCs, cancer stem cells; EMT, epithelial to mesenchymal transition; HDAC, histone deacetylase; KDM1, lysine-specific demethylase 1; MDR, multidrug resistance; RTK, receptor tyrosine kinase; VEGF, vascular endothelial growth factor.

Over the past few decades there have been many strategies used to treat cancer, from conventional radiation therapy, chemotherapy to recent targeted therapy, and immunotherapy. It is no exaggeration to say that the flow of this change has been led by the discovery of various factors that cause MDR acquisition in cancer cells, especially ABC transporters. By discovering ABC transporters and identifying their functions, new possibilities have emerged for cancer treatment. Initially, many studies were focused on the direct inhibition of ABC transporters such as P-gp inhibitors. However, because they are less selective and less potent, differ in their *in vitro* and *in vivo* data, and often cause severe adverse effects, so far no drugs that directly target or inhibit P-gp have been accepted for clinical use. Although direct inhibition of ABC transporters may not be effective, the transporters remain attractive targets because enhanced drug efflux through the transporters is one of the most important causes of MDR acquisition. Therefore, rather than directly inhibiting the ABC transporters, it would be effective to devise alternative strategies to avoid drug efflux via transporters. The transporter-mediated MDR might be overcome by developing novel anticancer drugs with P-gp non-substrates. In addition, we have introduced alternative approaches for targeting CSCs that focus on ALDHs, EMT, epigenetic modifications, the microenvironment, and signaling pathways (Figure 1). We have also discussed the drugs being developed in each approach: (i) ABC transporter inhibitors and non-substrates; (ii) ALDH inhibitors; (iii) AMPK activators; (iv) BET inhibitors, HDAC inhibitors, and KDM1 inhibitors; (v) VEGF inhibitors, RTK inhibitors, and vascular disrupting agents; (vi) Notch inhibitors, Smo inhibitors, and Wnt/β-catenin inhibitors. One of the big obstacles we are facing now is whether we can selectively influence CSCs only. NSCs and CSCs share the same characteristics; therefore, solving this problem is a complex task. Although promising solutions have been proposed, more research should be conducted to support the arguments relating to nanotechnology-based drug delivery, RNAi technology, and gene transfer technology. It is worth noting that these approaches are directly or indirectly related to ABC transporters. It is hoped that all of the approaches reviewed here will help devise new strategies to overcome MDR and to eradicate MDR cancer.

## Author Contributions

YC and YK participated in manuscript drafting and revision of this article.

## Conflict of Interest

The authors declare that the research was conducted in the absence of any commercial or financial relationships that could be construed as a potential conflict of interest.
